# Insulin and Diabetes

**Published:** 1923-01

**Authors:** 


### INSULIN AND DIABETES.

### THE TYPES FOR WHICH CURE IS IN SIGHT.

THE news that a cure for diabetes has at last been discovered must have brought relief to many anxious minds. Fortunately, it seems that on this occasion the discovery is genuine, and that future disappointments are not in store. Relief for thousands of sufferers may be really in sight.

Nevertheless, before we can apply this happy prediction to the individual case, it is essential to realise that diabetes as commonly understood—namely, of the passage of sugar in the urine—is not a disease in itself. It is only a *sign of disease,* in the same way that giddiness or back-ache might be. The seriousness of the case and the future outlook of the patient depend entirely upon the underlying condition, and not upon the fact that he does or does not pass sugar. In brief, there are several kinds of diabetes, and their outcome varies from moderate personal inconvenience to invariable fatality.

It may be said straight away that Insulin aims to deal with the severe type; but a few instances of these different grades will make the point clearer, and show also exactly when and how this new cure may be expected to succeed.

### How Sugar is Used in the Body.

When sugar is taken as food, it is rapidly absorbed into the circulating blood, which then conveys it to all parts of the body for storage or immediate use as the need may be. After a typical meal, most of the sugar is stored in the liver, some in the muscles, and the rest (a very little usually) remains free to be utilised then and there as body fuel.

Exactly how much is stored depends upon a very important circumstance. Sugar is a direct food to all the muscular tissues. In the blood of a healthy person the sugar content is always at practically the same level. When, by movement, etc., we begin to use the sugar up, the loss is constantly being made good from the store. Therefore two conditions are necessary to health: (1) The processes by which sugar is packed away in the liver and muscles must be working properly, and (2) the amount of sugar in the blood must be kept at the proper amount.

### The Severe but Rarer Disease.

Now upon these two conditions we can build a simple picture of diabetes. Let us take the severe type first. The storage process, as might be imagined, is highly complicated and involves intricate chemical changes. The changes, as with most of our internal mechanisms, are kept going by certain *enzymes* or special substances passed into the blood by the glands as it flows through them, and more familiar, perhaps, under the name of "internal secretions." If these substances are absent or ineffective, the sugar will not be stored properly. That is to say, although the person has eaten and absorbed into his blood an apparently satisfactory amount of carbohydrate (sugar), yet the storage chambers will fail to take it up. The resulting excess of sugar in the blood is simply turned out by the kidneys. It appears in the urine and is wasted. The patient's body has not retained the sugar which it will later on require.

The result, unfortunately, is not simple starvation. His body tries to make up for its failure by utilizing all kinds of other substances—fats particularly—in an attempt to furnish the missing sugar. Loss of weight gradually occurs, but the abnormal use of fats also tends to produce a number of poisonous substances within the body. These slowly poison the nervous system, and the emaciated patient gradually drifts into that fatal sleep known as diabetic coma. The deficiency of one link in the chain has broken down the whole mechanism of food assimilation, and the toxic substances produced have completed the destruction.

### The More Usual Chronic Types.

It must not, of course, be imagined that the appearance of sugar in the urine usually heralds such disaster. Certainly in patients in the second half of life the mild forms are far commoner, and they often arise in quite different fashion.

For example, many of these people do not appear to need much sugar, and their bodies simply refuse to store it. The moment they take a little more than their immediate requirement, the excess is turned out through their kidneys. Their "sugar tolerance," as it is called, is low, but they may be in perfect health. They are troublesome from the insurance point of view, but no "cure" as such is really necessary.

Also a number of middle-aged folk, particularly the obviously stout and the heavy feeders, absorb far more sugar than they need. They store what is necessary and simply get rid of the rest. The urine contains sugar, but if the diet be reduced it rapidly disappears. The cure is a simple matter, and there is little need for apprehension.

Again, there are those whose natural filters, the kidneys, are not too satisfactory, and will allow sugar to escape too easily. A very slight rise in the sugar content of the blood will then cause its appearance in the urine. The cure of such people is cure of the kidneys, and has little to do with true diabetes.

So might one outline the stories of sundry other minor "diabetic" states. They are practically never fatal, and many are in truth curable by simple dietetic means. Certainly they cannot be classified with the true diabetes mellitus, the severe type, against which Professor Maclead and his colleagues in Toronto have now given us an effective weapon called Insulin.

### How Insulin Acts.

To realise the action of this substance we must, therefore, retrace our steps. It will be remembered that sugar storage in the normal person is controlled by an enzyme or internal secretion. It had long been thought that the glandular source of this enzyme was the pancreas, or, more accurately, certain parts of it known as the islets of Langerhaus. Nevertheless, no one had succeeded either in proving the fact absolutely or in extracting from mammalian pancreatic glands the particular active principle in a form that could, with effect, be given to patients in need of it. The discovery now announced is an achievement of both these things. The active extract has been named Insulin, and these observers have already collected considerable proofs that its administration will make good the deficiencies of severely diabetic patient. It must be realised, of course, that the last word has yet to be said. So far, for example, it has been possible to obtain success only when Insulin is *injected* hypodermically, and even then the dosage is not definitely determined, because there are indications that too high a dose might prove extremely dangerous. Also it is not yet known whether patients will need to continue this treatment for the rest of their lives, or whether a few injections will so stabilise their metabolism that the body adjusts itself anew.

It is certainly clear that Insulin treatment, even if supplies were available, has not yet reached the stage when it can be presented to the profession for general application. The therapeutic difficulties have yet to be solved.

Nevertheless, it seems justifiable to assume that, under the guidance of the Medical Research Council, a specific extract for the relief of true, fatal diabetes will soon find its place in our pharmacopœias. A few months hence the matter will be actively tried out in our own great hospitals, and though for the moment this treatment cannot be called up at will, yet its unqualified success in every-day medicine will not, we believe, be very long delayed.

